# Recent Advancements in Acrylic Fabric Applications: A Comprehensive Review and Future Trends

**DOI:** 10.3390/polym16152111

**Published:** 2024-07-24

**Authors:** Raphael Palucci Rosa, Giuseppe Rosace, Valentina Trovato

**Affiliations:** 1 Department of Engineering and Applied Sciences, University of Bergamo, Viale Marconi 5, 24044 Dalmine, BG, Italy; valentina.trovato@unibg.it; 2 Department of Engineering and Applied Sciences, University of Bergamo, and Local INSTM Unit, Viale Marconi 5, 24044 Dalmine, BG, Italy; giuseppe.rosace@unibg.it

**Keywords:** acrylic fibres, functionalisation, innovative technologies

## Abstract

Acrylic fibres, as synthetic polymers, have been used extensively in the textile industry to create a wide variety of products, ranging from apparel and home furnishings to car rooftops and carbon fibres. Their widespread application is attributed to a combination of desirable properties, including a soft, wool-like texture, chemical stability, and robust mechanical characteristics. Furthermore, the chemical structure of acrylic fibres can be modified to imbue them with additional features, such as antimicrobial properties, fire resistance, conductivity, water repellency, and ultraviolet protection. This review explores the technological methods employed to functionalise acrylic fibres and discusses future trends in their development.

## 1. Introduction

**Figure 1:**
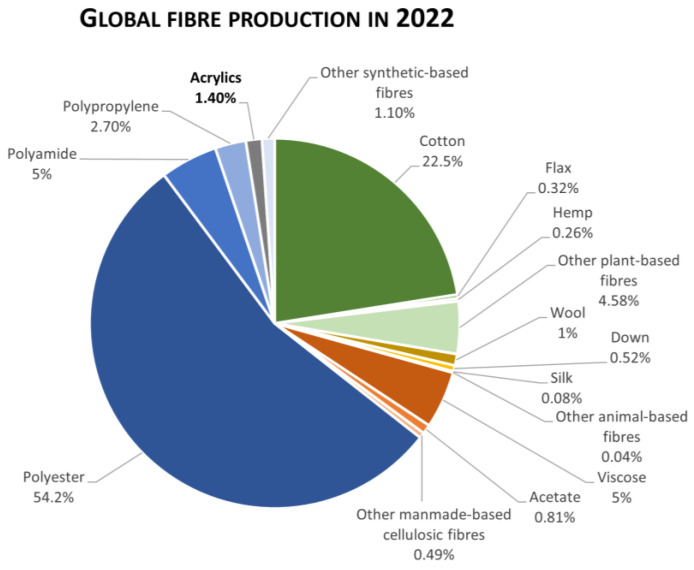
Global fibre production in 2022. Total acrylic fibres production is in bold. Data adapted from Textile Exchange [1].

The global market of textiles in 2022 was valued at USD 1695.13 billion, with polyester being the most produced material, accounting for approximately 54%, followed by cotton (22%) and polyamide (~5%) [1]. Among the synthetic polymers, acrylic fibres, also known as polyacrylonitrile fibres (PAN), “Aulon”, and/or “Cashmiron”, are widely used in the textile industry. Originally created by the DuPont Corporation in 1941 under the trademark “Orlon,” acrylic fibre is now used in the fabrication of a wide range of products, such as clothes, blankets, apparel, carpets, home furnishings, awnings, hand knitting and craft yarn, and stuffed toys [2]. As shown in Figure 1, in 2022 acrylic fibres accounted for 1.4% of the total global fibre market and 2.1% of the synthetic fibre market, with a global market share of USD 23.73 billion [1,3]. Moreover, it is the third most recycled synthetic fibre, where it is estimated that 50% of the total acrylic market share was derived from recycled fibres [1]. Furthermore, because of the low cost and versatility, it is anticipated that the global acrylic fibre market will grow by 46.4% by 2033, with significant demand coming from the Asia–Pacific region, the Middle East, Africa, and Latin America [4].

Although other fibres have an economic advantage over acrylic fibres because of better raw material availability, large-scale production, and recyclability, in the awning and outdoor furnishing textile market, acrylic fibres remain the predominant material (accounting for more than 90% of production), thanks to their unmatchable performance and properties [5]. Acrylic fibres have shown wool-like characteristics, like being soft when handled, warm, and lustery [6]. They also have demonstrated minimal water absorption, good ultraviolet (UV) light exposure tolerance, and average elongation at break and tensile strength compared to polyester fibres [7,8,9]. Moreover, they are resistant to a wide range of chemicals and can effectively endure acids and mild alkalis. However, exposure to strong alkalis at high temperatures can lead to rapid deterioration [7].

**Figure 2:**
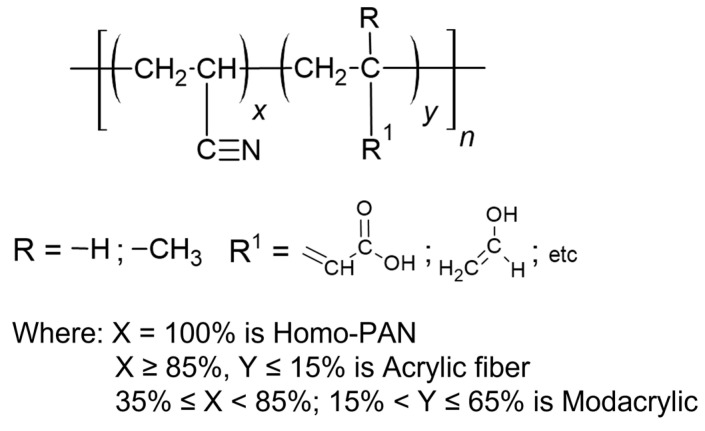
Acrylic fibre structure.

Normally, acrylic fibres are mainly synthesised by the polymerisation or copolymerisation of acrylonitrile and other vinyl monomers, such as acrylic acid, vinyl alcohol, vinyl chloride, vinyl acetate, vinylidene chloride, methacrylic acid, and methacrylate esters, with a variable degree of polymerisation (Figure 2) [10,11]. Although PAN fibres can also be synthesised through anionic photopolymerisation, the radical-initiated polymerisation or copolymerisation method is the predominant method of production [12]. According to the International Synthetic Fiber Standardization Office (BISFA), a fibre can only be defined as “acrylic fibres” if the polymer matrix has at least 85% of acrylonitrile units (–CH_2_–CH(CN)–). If the acrylonitrile content is between 35 and 85%, then the fibres are denominated as modacrylic, which can be used to add specific properties to the textile (e.g., dyeability) [13]. On the other hand, fibres made exclusively from acrylonitrile are considered homopolymer polyacrylonitrile (homo-PAN), typically used in the production of carbon fibres and filtration systems [14,15]. In all three types of acrylic fibres, the carbon-nitrogen triple bond is a distinctive feature that allows for their identification in the range between 2240 cm^−1^ and 2260 cm^−1^, distinct from other fibres, using IR spectroscopy [16,17].

Most acrylic fibres have number and weight average molecular weights of 40,000–70,000 g/mol and 90,000–170,000 g/mol, respectively, a density of 1.16–1.18 g/cm^3^, are soluble in polar aprotic solvents, and possess moderate strength and elongation at break. Their elongation at break can vary between 20 and 50%, depending on the fibre composition; however, at two percent elongation, the fibre recovery is 99%, while at five percent, it drops to 50–95%. Nevertheless, these fibres have shown excellent resiliency and recovery from bending deformation. Dry acrylic fibres have a tenacity that varies from 2 to 4 g/d (18–36 g/tex), which decreases to 1.5–3 g/d (13–27 g/tex) on exposure to moisture. They also have a moisture regain of 1.5–2% (at 25 °C and 65% relative humidity). Finally, these fibres have demonstrated good electrical and heat insulation properties [18].

Textiles are produced from acrylic fibres through two main methods: wet and dry spinning. The first method, wet spinning, is a low-temperature process generally used to fabricate unsuitable fibres for melt spinning [19]. This method stands out for its ability to create fibres of varied cross-sectional shapes and sizes [20]. During this process, a polymeric solution is extruded through small orifices of a spinneret into a coagulating bath. This bath effectively removes the solvent, solidifying the polymer filaments. The fibres are then collected and merged to form a continuous strand or rope [21,22]. The success of fibre formation in the wet spinning of acrylics is influenced by several factors, including the composition of the coagulation bath, the extrusion rate, temperature, solubility, take-up velocity, and spinning pressure [20,23].

The second method, dry spinning, is normally used to produce textiles requiring durable, wear-resistant fibres, such as carpets [24]. This process begins by dissolving the polymer using an organic solvent, such as acetone or ether. Following this, additives are mixed into the solution to create a viscous mixture referred to as “dope”. The dope is then filtered, de-aired, pre-heated, and transferred to an evaporation chamber, also known as a spinning tube, and is kept there awaiting extrusion. During extrusion, the dope is forced through fine openings (spinneret or jets) at a controlled rate. At this stage, the polymer solution is exposed to a stream of hot gas, which evaporates the solvent and increases the polymer concentration, thereby solidifying it. The resulting solid filaments are then collected by rotation rolls and put onto bales (or bobbins). Alternatively, the filaments may be further processed through stretching, twisting, or combining with other fibres, such as cotton or wool, to enhance their properties or functionality [25,26,27,28].

Depending on their intended application, acrylic fibres may undergo a process commonly referred to as textile finishing, wherein their chemical structure is functionalised to enhance or introduce desired properties. These properties may include improved dyeability, electrical conductivity, fire resistance, water repellency, antimicrobial activity, mechanical strength, and other characteristics not inherent to neat polymers [29]. However, this makes the management of acrylic textile waste a challenge. The EU-funded REACT (REcycling of Waste ACrylic Textiles) project aimed to identify possible processes to treat and recycle textile waste in an ecological and economic way [30]. Therefore, there is a strong need for innovative, high-quality products that are designed, engineered, and processed to meet specifications for different economies and societies; this includes rethinking the raw materials, processes, and services involved as well as investing in technology. The increasing attention of consumers regarding environmental questions is also creating a positive context for new recycling chains.

This review focuses on applying recent methods for acrylic fabric finishing, using surface modifications by plasma or chemical grafting, the sol-gel technique, layer-by-layer deposition of thin films, and other aqueous methods. As a result, the newly developed finishes based on nanoscience appear as valuable tools that can project an enhanced image of chemicals for the next textile industry revolution. Thanks to the comprehensive scheme of this review, it is possible to deduce that the use of novel and environmentally friendly finishes to produce durable and/or high-performance products, as well as more easily recyclable PAN or its use in the conversion of polymers into oligomers or monomers for specialty chemical production, seems the most promising for the future use of acrylic fabrics on the market.

## 2. Functional Finishing of Acrylic Fibres

This section explores the various functionalisation techniques for acrylic fibres by focusing on enhancing properties, such as durability, water repellency, flame retardancy, and antimicrobial resistance. In particular, we show how these modifications improve textile performance and open new application possibilities by highlighting the intersection of technology and textile innovation.

### 2.1. Antimicrobial Fibres

With the passage of time, antimicrobial and antifungal properties have become crucial for all types of textiles. Not only do they prevent material degradation, they also offer protection against the spread and transmission of pathogenic microorganisms, which has become of paramount concern for public health and safety in the post-COVID-19 landscape [31,32]. The pandemic underscored the importance of materials that can effectively inhibit the growth and spread of viruses and bacteria, highlighting the need for textiles with enhanced antimicrobial and antifungal capabilities in various applications, from medical settings to everyday clothing and home furnishings [33,34]. Specifically, acrylic fibres can be enhanced with various finishing agents to improve their protective capabilities, as shown in Table 1. For instance, El-Sayed et al. demonstrated that acrylic fibres could be combined with a polyurethane–chitosan solution, using hydrazine hydrate to increase its uptake by the fabric. The resulting modified acrylic fabric exhibited superior bacterial resistance compared to its untreated counterpart, maintaining its effectiveness even after 15 washing cycles [35].

Silver has long been recognised for its effective antimicrobial properties against viruses, fungi, and most bacteria [36,37]. Due to its properties, it has also been tested for modifying textiles. Yu et al. used a dry jet–wet spinning technique to produce a silver-loaded hollow fibre. Since the silver was inside the fibre, it was noted that the fabric retained its silver content even after being washed with water for over 60 days, with only a 0.1% reduction in the silver. Furthermore, the acrylic acted as a barrier, releasing the silver particles and thereby continuously replenishing its antimicrobial properties [38]. Allehyani et al. functionalised acrylic fabric with amidoxime groups to act as ligands for the immobilisation of silver ions. The functionalised fabrics showed good tensile strength and excellent antimicrobial properties, so much so that, in the latter case, the AgNP-loaded acrylic fabric completely eliminated *E. coli* after 24 h [39]. Wang et al. incorporated silver nanoparticles (AgNPs) into PAN fibres during the electrospinning process. Their findings revealed that the PAN fibre with three percent AgNPs exhibited remarkable antibacterial effectiveness, achieving a 99.99% reduction rate for both *E. coli* and *S. aureus* [40].

Other types of antimicrobial materials and polymers have also been tested in acrylic fabrics. Lee et al. combined poly(styrene hydantoin) (PSH) with PAN using a wet spinning process. The resulting fabric was then soaked in a commercial sodium hypochlorite solution for 30 min to imbue it with antibacterial properties. Their findings indicated a direct correlation between chlorine concentration and antibacterial efficiency. Moreover, the functionalised acrylic fibres retained their protective properties even after 50 washing cycles, showcasing the durability of the antimicrobial treatment [41].

**Table 1:** The effectiveness of various antimicrobial treatments on *E. coli* and *S. aureus*.

Author	Material Used	Inhibition Zone Diameters (mm)		Ref.
		*E. coli*	*S. aureus*	
Houng et al.	Silver/graphene oxide (Ag/GO) nanocomposite	-	24.00 ± 0.34	[37]
Okay et al.	Silver nanoparticles, acrylic acid, and acrylamide	10.25 ± 0.35	11.00 ± 1.41	[42]
Patel et al.	Zinc oxide and silver nanoparticles	10.00	-	[43]
Mofidfar et al.	Poly(acrylic acid) fibres with silver nanoparticles	-	3.00 ± 0.3	[44]
Chen et. al.	Tannic acid and silver nanoparticles	11.00 ± 0.5 ^a^	11.60 ± 0.5 ^a^	[45]
Sarwar et al.	Diclofenac Sodium Salt	16 ± 0.46	15.5 ± 0.28	[46]
Wahab et al.	Titania/AgNP composite nanoparticles	3.23	4.1	[47]

^a^ Approximate value.

### 2.2. Electronic Textiles

Electronic textiles, commonly known as e-textiles, are fabrics or textile materials capable of conducting electricity; alternatively, electronic components can be embedded in their structure [48]. These textiles can be used in many applications, such as creating protective clothing that shields against electromagnetic radiation [49,50], developing wearable sensors for health monitoring (glucose, electrocardiography, dehydration) [51,52,53], measuring pressure and humidity [54,55], and producing energy storage and generation devices, such as solar cells and supercapacitors [56]. The surge in the popularity of e-textiles can be attributed to two main factors: First, the unique properties of textile materials, such as stretchability, foldability, and porosity, provide the ideal conditions for supporting electronic devices. Second, integrating such devices with the Internet of Things can enable rapid analyses of the data from these devices, providing the user with real-time information [57,58].

Through the electrical or electronic integration method, e-textiles can be categorised into three main types [59]. The first type combines conventional electronic components, such as conducting wires, integrated circuits, LEDs, and batteries, into garments. For reasons of simplicity and low cost, this is the most frequently used type of integration [60,61]. The second type integrates electronic components directly into textile fibres, including both passive (e.g., wires or conducting fibres) and active (e.g., transistors, diodes, and solar cells) elements [62,63]. The third category is the most advanced form of integration, where the textile fibres themselves function as sensors or actuators. In this approach, the fibres are designed or functionalised to possess inherent sensing or actuating capabilities, making them an integral part of the e-textile system [48].

There are various approaches to coating acrylic fibres, fabrics, and textiles with conductive materials (Table 2) [64]. For instance, Baseri et al. investigated the coating of PAN with copper sulphide in order to add electrical conductivity to it. The authors studied how the concentration of copper (II) sulphate, temperature, and the ratio of reducing agents influenced coating conductibility performance. According to them, the mixture containing 1.2 g/dm^3^ of copper (II) sulphate, 3.6 g/dm^3^ of sodium thiosulfate, 1.6 g/dm^3^ of hydroxylamine sulphate, and 3.0 g/dm^3^ of sodium dithionite showed the best results. The resistance of the neat acrylic decreased from 10^12^ O per square to 10^2^ O per square. The authors also found that when the reaction was performed at higher temperatures (85 °C), the acrylic fibres became more porous, resulting in better copper attachment. However, increasing the temperature above this point showed no significant improvement in the conductivity of the fibre [65].

Ahn et al. explored enhancing the electrical conductivity of PAN fibres using neat and modified carbon black nanoparticles (CB). The latter had its structure modified using 4-aminobenzoyl, benzoxazine, and Ag metal particles. The results showed that when neat CB and its modification were combined with PAN fibres, conductivity and tensile strength increased drastically in all cases. In particular, the fibres containing CB and benzoxazine achieved a tensile strength and conductivity of 110.4 MPa and 8.9 × 10^−4^ S/cm, respectively [66]. Kayabaşi et al. also conducted tests using CB to decrease the electrical resistance of different textiles, including acrylic fibres, by integrating CB into the fibrous structure before yarn and fabric production. This was done to maintain the softness, breathability, and flexibility of the textiles, improving their wearability. As reported by the authors [67], when 0.5 wt.% of CB was impregnated into the acrylic yarns, the sample resistance decreased to approximately 1000 kΩ. However, the resistance increased between 1.5 and three times after the yarns were washed. Furthermore, the results showed that the incorporation of CB did not affect the tenacity and elongation of the mechanical characteristics of the materials at break.

**Table 2:** Conductivity analysis of various nanomaterials.

Author	Material	Amount	Final Conductive (µS/cm)	Ref.
Ahn et al.	Carbon black nanoparticles	12%	890	[66]
Rehan et al.	Silver nanoparticles	0.05%	324 ± 0.5	[68]
Karbownik et al.	Polyaniline fibres	1%	100	[69]
Mustafov et al.	Lignin with graphite	20%, 1 wt.%	19.2 × 10^3^	[70]
Xi et al.	Poly (3,4-ethylene dioxythiophene) (PEDOT)	150 µL/10 mL	20.51 × 10^6^	[71]
Deng et al.	Multi-walled carbon nanotubes (MWCNTs)	6 wt.%	51.6 × 10^6^	[72]
Mpukuta et al.	Silica nanoparticles	1 wt.%	8.11 × 10^3^	[73]

### 2.3. Flame Resistance

Acrylic fibres are known for their high flammability, characterised by a limiting oxygen index (LOI) of 18, which places them in the category of materials with a potential fire risk [74,75,76]. Consequently, there has been a significant effort to develop efficient flame retardants (FRs) for them, particularly between the 1950s and 1980s—often referred to as the golden era of FR development. These retardants mainly consisted of halogens, organohalogens, and formaldehyde-based products [77,78]. However, due to the high toxicity of these materials and their combustion by-products, most halogenated and formaldehyde-based FRs have been phased out or completely banned in recent years. This led to companies to invest in the development of alternative products, like the Kanecaron^®^/Protex^®^ (produced by Kaneka), which not only increased the LOI of treated PAN fibres to 31 but also conferred them high chemical resistance against acid and alkali [79,80]. Additionally, there has also been a surge in academia in the research and development of less hazardous and more environmentally friendly FRs in recent years (see Table 3) [81].

Zhou et al. enhanced the flame resistance of PAN fibres by introducing sodium ions via a post-treatment process involving hydrazine hydrate (HHA) and a sodium hydroxide solution (NaOH). The fibres underwent testing through sequential immersion in two solutions at 95 °C: first, a 40 wt.% aqueous solution of HHA, followed by a 15 wt.% aqueous sodium hydroxide solution, each for varying durations. The PAN fibres immersed for 60 min in HHA and 30 min in the NaOH solution showed an LOI increase of 163% (from 19 to 60), with the residue from the Na-PAN combustion acting as a protective barrier against further combustion. However, submerging PAN fibres for longer than 30 min in HHA embrittles the fibres, reducing their tensile strength [82]. To counteract this and improve the mechanical properties of the PAN fibre, Zhou et al. continued by reinforcing acrylic fibres with poly(vinyl alcohol) (PVA). The addition of PVA doubled the tensile strength of the fibres, increasing from 1.96 to 4.01 cN/dtex. However, the Na-PAN LOI decreased by 51.5% to 29.1, although its value remained above what is considered combustible (LOI ≤ 21) [83,92].

Using another approach, Carosio et al. coated acrylic fabrics by alternating layers of ammonium polyphosphate and octapropyl ammonium polyhedral oligomeric silsesquioxane. They created a fabric with a total of 12 layers, suppressing melt dripping and proving effective against a 35 kW/m^2^ heat flux. Additionally, its LOI increased by 10%. Although the LOI increase was modest (from 20.5 for the neat acrylic to 22), the treated fabric retained 40.7% of its mass after burning tests, significantly outperforming the untreated acrylic, which was completely consumed [84,93].

**Figure 3:**
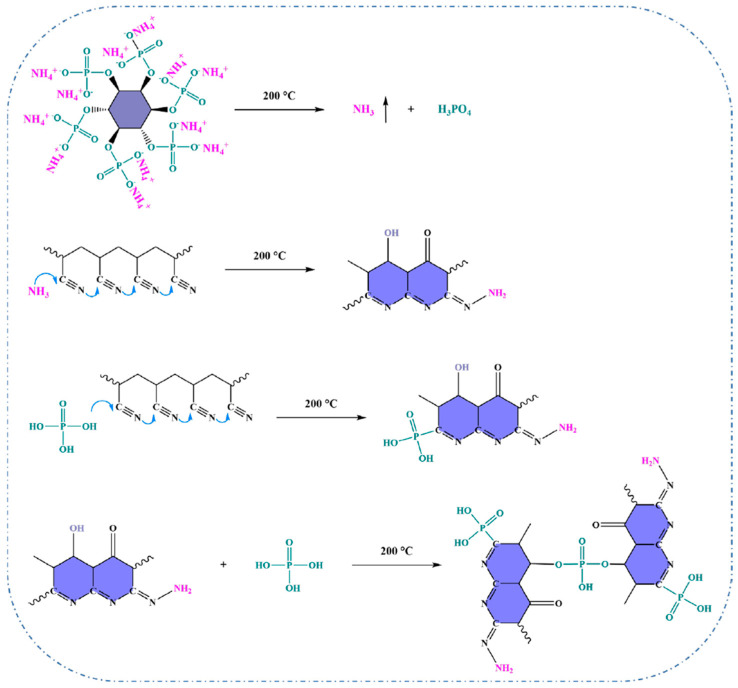
Possible formation mechanism of O-AP-PAN fibres. Reproduced under the terms of the Creative Commons CC BY-NC-ND 4.0 license. Copyright 2023, the Authors [91]. Published by Elsevier.

More recently, Zou et al. produced FR PAN fibres (O-AP-PAN) by immersing the PAN fibres in an ammonium phytate (AP) solution, which was prepared by neutralising phytic acid with urea, followed by a thermal oxidation treatment (Figure 3). The treated fibres demonstrated an increase in LOI value to 36.6% and significant reductions in total heat release and heat release capacity by 39.9% and 52.7%, respectively. Furthermore, the fibres maintained their FR characteristics even after 50 wash cycles, with a post-wash LOI value of 30.5%. When the fibres were subjected to fire, the O-AP-PAN fibres generated a stable layer of graphitised carbon, effectively preventing the inner fibres from being exposed to oxygen and heat [91].

### 2.4. Water Repellency and Waterproof Finishes

Textiles with water repellency are beneficial for numerous applications, ranging from simple waterproof cycling suits to personal protective equipment that shields against hazardous liquids [94,95]. The key to water repellency is based on the surface tension value of the fabric, which does not allow the fabric to become wet as long as it is lower than the surface tension of water. Common finishing chemicals, such as silicones, fluorochemicals, wax, and resins, are used to add or improve the water repellency of textiles [96].

However, over the last decade, it has been discovered that fluorocarbon-based compounds, in particular, perfluorooctane sulfonate (PFOS) and perfluorooctanoic acid (PFOA), are a serious threat to the environment and a health risk to humans due to their bio-accumulative and toxic nature [97]. Therefore, several governmental regulatory agencies around the world have imposed restrictions on their usage. An immediate measure was to shorten the fluorocarbon chain length from C8 to C6 or C4, mitigating environmental and health impacts; at the same time, this has resulted in the loss of FR efficacy. This challenge has prompted research into new techniques and materials (as shown in Table 4) to either improve or replace fluorocarbon-based compounds [98].

Ceria et al. used atmospheric plasma treatment as a preliminary step to improve the durability of the conventional pad-dry-cure finishing method for acrylic fabrics. The finish consisted of a mixture of perfluoroalkyl acrylate copolymer emulsion and methoxymethyl melamine resin as a crosslinking agent. They found that the plasma-treated samples performed better than the untreated samples in maintaining their water repellency property after 50 washing cycles. Initially, both the treated and untreated samples achieved a water repellency grade of 8, but this decreased to 4.5 for the untreated and 6.5 for the treated samples after repeated washing [99].

Li et al. proposed a one-step fluorine-free method to fabricate waterproof and breathable fabrics. First, PAN and blocked isocyanate prepolymer (BIP) were combined using an electrospinning technique, which allows the manufacturing of fibrous membranes with controllable fine fibre diameter, stacking density, and ease of surface modification. Following the production of the fabrics, they were dip-coated with a hydroxyl acrylic resin (HAR) emulsion, and their water repellency was evaluated. Optimal results were obtained when the PAN/BIP fibrous membranes were coated with two percent of HAR. Initially, the PAN/BIP membranes had a water contact angle of about 60°, which increased to 151° (indicating high hydrophobicity) post-coating. Furthermore, the PAN/BIP membranes with two percent of HAR also demonstrated a tensile strength of 12.3 MPa, a waterproofness of 112.5 kPa, and a water vapour transmission of 12.7 kg m^−2^ d^−1^, performance levels comparable to those of high-end fluorinated finishes [100].

**Table 4:** The effectiveness of different water repellency methods on acrylic-based textiles.

Author	Materials/Method	Amount	Final Contact Angle	Water Vapour Transmittance (kg m^−2^ d^−1^)	Hydrostatic Pressure (kPa)	Ref.
Li et al.	Blocked isocyanate prepolymer (BIP) and fluorine-free waterborne hydroxyl acrylic resin (HAR)	2 wt.% of BIP and 2% of HAR	151°	12.7	112.5	[100]
Wang et al.	Polyurethane (PU) and silicon dioxide nanoparticles (SiO_2_)	50 wt.% of SiO_2_	151.2°	10.8	85.7	[101]
Zhang et al.	Amino functional modified polysiloxane (AMP) and 4, 4′-methyl diphenylene diisocyanate (MDI)	1 wt.% of AMP and 2 wt.% of MDI	139.2°	4.7	93.8	[102]
Gu et al.	Polyvinylidene fluoride (PVDF)	3 wt.%	137°	4.65	18.04	[103]
Yu et al.	PU and SiO_2_ nanoparticles	3 wt.% of SiO_2_	137.2°	10.3	-	[104]
Liu et al.	(3-aminopropyl) triethoxysilane (APS) and hexadecyltrimethoxysilane (HDTMS)	10 wt.% of APS	150.1°	3.92	-	[105]

### 2.5. UV Protection

UV radiation is non-ionising radiation emitted by the sun and artificial sources, such as UV lamps. UV radiation can be divided into three types: UV-A (320–400 nm), UV-B (290–320 nm), and UV-C (200–290 nm), but the latter does not reach Earth [106]. While moderate UV light exposure has shown health benefits, such as being partly responsible for producing vitamin D in organisms, prolonged exposure can lead to skin cancer. Therefore, developing textiles with UV protection offers a valuable means to shield human skin from excessive exposure to UV-A and UV-B rays, minimising the potential for skin damage [107,108].

Titanium dioxide is widely used to add UV-blocking properties to acrylic fibre (Table 5). It is very attractive for the functionalisation of textiles due to its chemical stability at high temperatures, nontoxicity, and permanent stability under UV light [109]. Accordingly, Nazari et al. evaluated the performance of acrylic fabrics coated in a solution of TiO_2_ nanoparticles and polysiloxane. The optimal concentration of TiO_2_ and polysiloxane was defined as 0.68% and 2.19%, respectively. The acrylic fabrics treated with nano TiO_2_/polysiloxane not only absorbed light with a wavelength lower than 400 nm, but their self-cleaning and soft handling properties were also enhanced [110].

Silver, commonly used to add antimicrobial properties, can also provide UV protection to treated fabrics (Table 5) [111]. Hassan et al. produced multifunctional acrylic fibres by coating them with Ag particles at varying concentrations. It was observed that UV protection increased according to the Ag concentration. When three percent of Ag was applied, the UV transmission through the fabric reduced from 17.72% (undyed acrylic yarns) to 0.6%, a 97% decrease. Furthermore, the fabrics also showed good antistatic and antimicrobial properties [107].

El Gabry et al. employed eco-friendly methods to functionalise acrylic fibres for UV protection, moisture retention, and air permeability. They employed the pad-dry-cure technique using sodium polyacrylate/bentonite nanocomposites to modify the fibres. Thereafter, nanoclays, such as nano bentonite and its nanocomposites, were used as a coating. This treatment boosted the ultraviolet protection factor (UPF) from 9 to 36, a substantial increase, indicating excellent protection against UVA and UVB rays. The authors suggested that the bentonite nanocomposite acted as a barrier, blocking UV radiation from penetrating the fabric [29]. Singh A. and Sheikh J. evaluated the usage of ethyl anthranilate to create a dye for acrylic textiles to add UV protection and mosquito repellency. The fabrics dyed with 3 wt.% of ethyl anthranilate had an increase of 1358% in their UV protection (from 18.72 to 272.95) while showing a mosquito repellency of 97.67% [112].

**Table 5:** Types of materials and their influence on UPF and UV protection.

Author	Material	Amount (%)	UPF	UV Protection Improvement (%)	Ref.
Hassan et al.	Silver nanoparticles	3	-	94.8	[107]
El Gabry et al.	Sodium polyacrylate/bentonite nanocomposite	-	36	300	[29]
Rehan et al.	Silver nanoparticles	0.05	541	1400	[68]
Jiang et al.	Titanium dioxide (TiO_2_) nanoparticles	10	175	-	[113]
Koozekonan et al.	Titanium dioxide nanoparticles	15	133	1425	[114]
	Carbon nanotubes (CNT)	10	48	450	
	TiO_2_ with CNT	15	685	7755	
Nasouri et al.	Multi-walled carbon nanotubes (MWCNTs)	10	677	-	[115]

## 3. Recent Technologies in Acrylic Textile Finishing

This section explores the advancements in acrylic textile finishing and focuses on plasma treatment, sol-gel techniques, and bio-based finishes. These technologies not only improve fabric performance but also help the textile industry shift towards more sustainable and environmentally friendly practices.

### 3.1. Plasma Treatment

Plasma technology offers a dry, solvent-free, environmentally friendly, and worker-safe alternative for modifying the surface of textiles and requires minimal energy and time [116]. Unlike other textile processes, plasma modification is limited to the first atomic layers of the fibre surface and does not change the properties of the whole fibre. Atmospheric cold plasmas are usually used for textile finishing processes, as most of them are sensitive to heat [117]. Additionally, as it is more environmentally friendly than other traditional methods and is driven by new technological advancements, its adoption has been growing in recent years [118,119].

Plasma is a mixture of activated ionised gases used to modify the surface and coating of textiles [117]. It utilises a variety of gases, including polymerisable, non-polymerisable, and chemically inert types, such as nitrogen (N_2_), argon (Ar), and helium (He), to induce a range of properties in textiles. These include surface activation, oxidation, modification of surface energy, coatings, alteration of roughness, and the creation of micro/nanostructures [116].

Plasma treatment has been demonstrated to be an effective method to enhance the water repellent properties of acrylic fibres [99,120] and improve the dyeability of natural dyes [121]. In the first case, Pane S. et al. developed a viable method for cleaning and coating fabric surfaces, achieving outcomes comparable to those of traditional approaches [120]. Moreover, the process was also able to improve the durability of fluorocarbon finishes over long washing cycles [99]. In the second case, Haji A. et al. used plasma treatment to improve the dyeability of acrylic fabrics by grafting amide and carboxyl groups onto their surface. The introduction of these polar groups increased moisture penetration, reduced the fibre contact angle, and enhanced the surface energy and surface roughness [121].

**Figure 4:**
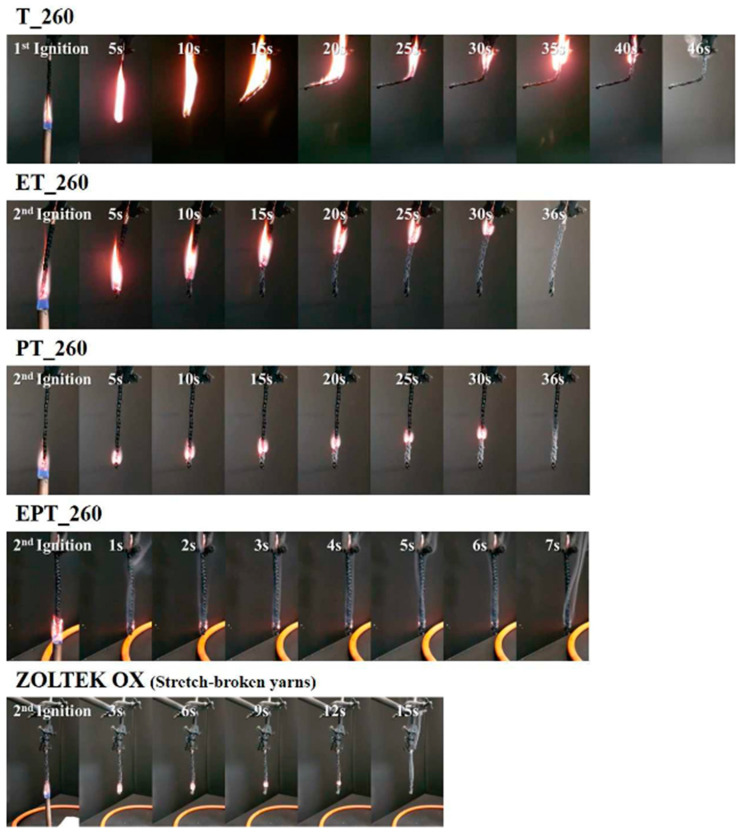
Combustion processes of T_260, ET_260, PT_260, EPT_260, and ZOLTEK OX during the UL 94 vertical burning test at different times. Reproduced under the terms of the Creative Commons CC BY-NC-ND 4.0 license. Copyright 2019, the Authors [122]. Published by Elsevier.

Another application where plasma treatment has shown promising results is in enhancing the flame retardancy of PAN fibres. Kil H. and Lee S. demonstrated that plasma-assisted thermal stabilisation in combination with electron-beam irradiation can significantly improve the flame retardancy of PAN fibres. According to their results, the sample stabilised for 60 min (EPT_260) showed the best performance, with an LOI value of 50.9%. Furthermore, compared to the precursor PAN, the PHRR and THR of neat PAN (T_260) and EPT_260 dropped from 588 to 143 and 52 kW/m^2^, and from 35.4 to 16.9 and 7.3 MJ/m^2^, respectively. These results indicate a substantial enhancement in the flame-retardant properties, as shown in Figure 4 [122].

### 3.2. Sol-Gel

Sol-gel technology is another important system for functionalising textile materials, due to its cost-effectiveness, efficiency, reduced chemical usage, functional versatility, chemical stability, and minimal environmental impact [123]. The technology consists of creating an inorganic network through the formation of a colloidal suspension (sol) and a gelation process in a continuous liquid phase (gel) [124]. The resulting sol-gel solution is applied to the fabric and then heat-treated, forming a durable coating that can be combined with various organic and inorganic components [125].

The production of a sol-gel solution begins with the selection of suitable precursors in water or water-miscible organic solvents, such as ethanol or propanol. Subsequently, condensation reactions commence between the precursors, leading to the formation of an inorganic network [124]. When applied to textile substrates, the colloidal particles in the solution condense and aggregate, forming a wet layer that, upon evaporation of the solvent, leaves behind a ceramic film. The characteristics and mechanical properties of this film are greatly influenced by the parameters of the sol-gel process [124]. In recent years, sol-gel technology has become one of the most important types of chemical finishing, with the increased demand for innovative and environmentally friendly fabrics [123]. The method has been used to produce textiles with various properties, such as flame retardance, water repellency, self-cleaning, UV protection, and antibacterial activity. In the case of acrylic textiles, sol-gel has shown promising results in a range of applications [125].

**Figure 5:**
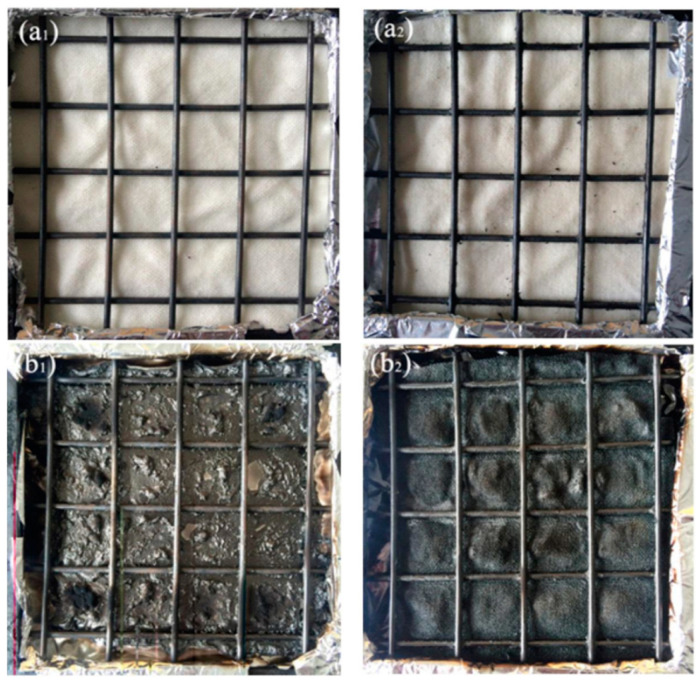
The digital photographs of the fabrics before and after CC testing. (**a1**) PAN fabric; (**a2**) FR-PAN fabric; (**b1**) PAN fabric after burning; (**b2**) FR-PAN fabric after burning. Reproduced under the terms of the Creative Commons CC BY 4.0 license. Copyright 2018, the Authors [126]. Published by MDPI, Basel, Switzerland.

Ren et al. investigated the FR finishing of PAN based on sol-gel technology. They first prepared a cationic silicon hydrogel solution using 3-aminopropyltriethoxysilane as a precursor and nitrogen source. Then, an anionic solution was prepared from phytic acid, which acted as the phosphorus source. The PAN fabrics were coated by alternating (10 times) silicon hydrogel and phytic acid solution and then dried in a vacuum oven at 60 °C for 30 min, followed by curing in the oven at 160 °C for 5 min. Their results showed that the coated fabrics possessed a LOI of 94% higher than the non-treated fabrics (increasing from 17 to 33). Moreover, as shown in Figure 5, after ignition, the FR-PAN formed a continuous and dense char layer across the fabric, which acted as an insulator blocking the transfer of heat and oxygen to the fabric, thereby preventing further combustion of the PAN fabrics [126]. Ying et al. tested the usage of tetraethyl orthosilicate (TEOS) to develop efficient oil-in-water emulsion membranes. To do this, PAN membranes, prepared via electrospinning, were coated with a TEOS-based silica sol-gel. To promote the condensation reaction, NH_4_OH was used. The PAN membranes were highly efficient for emulsion separation. For the toluene oil-in-water emulsion, the membranes achieved a 99.50% separation efficiency, while for the n-heptane, paraffin liquid, n-hexane, and xylene oil-in-water emulsion, the efficiency exceeded 99.68% [127].

### 3.3. Bio-Based Finishings

Bio-based polymers, which are derived from renewable sources, such as plants, microorganisms, fungi, and algae, have been gaining attention for their potential to reduce waste and dependence on non-renewable resources [128]. While the production of textiles from biopolymers is established, particularly in fabrics combining natural and synthetic fibres, the integration of bio-based and biodegradable materials into textile finishes is still being developed. Therefore, there is a high demand for bio-based and/or biodegradable finishing development [128].

Chitosan is a bio-based and biodegradable polymer with promising dye adsorption properties. It is derived from chitin, which is the second most abundant biopolymer on earth, and obtained from fungi, insects, and fishery waste [129]. Lou et al. explored the use of chitosan to improve the dyeability of Acid Blue 113 in PAN fibres. The finishing process consisted of a two-step process: creating a nanofibrous PAN mat via electrospinning and then immersing it in a chitosan solution, followed by 1 h in a vacuum to achieve a uniform coating. Using this process, chitosan homogeneously adhered to the PAN nanofibres, improving their hydrophilicity. The hydrophilicity improvement resulted in better dye adsorption, where the samples coated with chitosan achieved the maximal adsorption capacity of Acid Blue 113 (1708 mg/g) [130].

**Figure 6:**
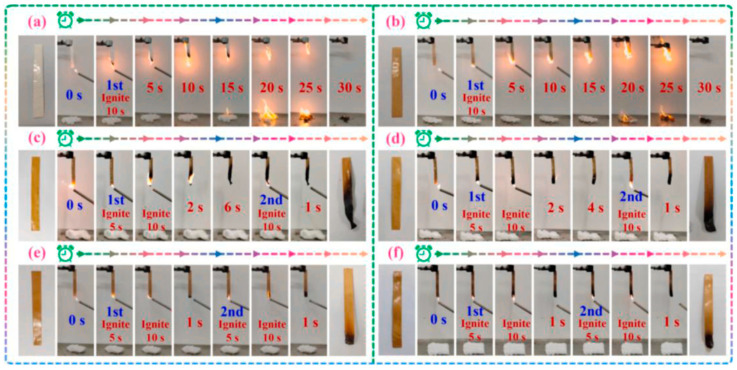
UL-94 test photos of PAN (**a**), L/C/PAN (**b**), FR-PAN-5 (**c**), FR-PAN-8 (**d**), FR-PAN-10 (**e**), and FR-PAN-12 (**f**). Reproduced under the terms of the Creative Commons CC BY-NC-ND 4.0 license. Copyright 2023, the Authors [131]. Published by Elsevier.

Other types of biomaterials were explored in the PAN fibres. For instance, Zou et al. tried to enhance the FR of PAN fibres by immersing them in an ammonium phytate solution and subsequently subjecting them to a thermal oxidation process. According to the results, fibres with a 5.4% weight gain had a significant improvement in their LOI, which increased from 17.0% to 36.6%. In addition, there was a substantial reduction in the total heat release and heat release capacity by 46.8% and 52.7%, respectively. Finally, it was also observed that even after undergoing 50 cycles of laundering, the LOI value of the treated fibres remained impressively high, at 30.5%, showcasing their superior washing durability [91]. In another work by the same research group, Guo et al. used lignin, casein, and phytic acid to improve the FR of the PAN fibres. Fibres treated with 10 wt.% (FR-PAN-10) and 12 wt.% (FR-PAN-12) of phytic acid demonstrated outstanding self-exhaustion properties (as shown by Figure 6), where the 12 wt.% samples had a 91.18% LOI increase (from 17% to 32.5%). Moreover, the modified PAN fibres also exhibited a remarkable reduction in both the peak heat release rate and the smoke production rate by 71.4% and 64.8%, respectively, demonstrating exceptional flame resistance and smoke suppression capabilities [131].

### 3.4. Grafting

**Figure 7:**
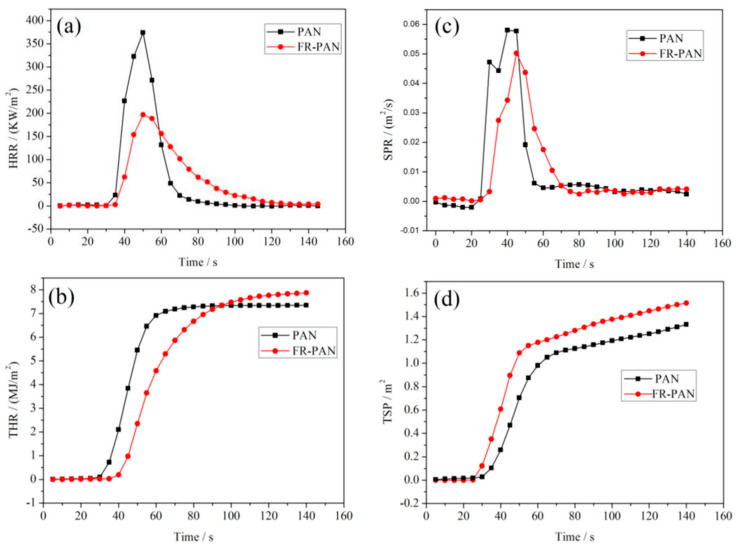
HRR (**a**), THR (**b**), SPR (**c**), and TSP (**d**) curves of PAN and FR-PAN fabrics. Reproduced under the terms of the Creative Commons CC BY 4.0 license. Copyright 2019, the Authors [134]. Published by MDPI, Basel, Switzerland.

The surface modification of PAN fibres through grafting techniques has been continuously explored as an interesting method to introduce different properties to them [132,133]. The process is usually performed by UV, since it is affordable, simple, and results in minimal to non-existent damage to the material structure [132]. For instance, Ren et al. used UV light to graft modified glycidyl methacrylate (GMA) into PAN fabrics in order to improve their fire resistance. They found that the treated fabrics had a 38.4% reduction in the total heat release (THR) rate and a 60.2% decrease in the peak heat release rate (PHRR). Furthermore, the treated fabrics achieved an LOI of 32.3%, remaining at 29.3% after 30 washing cycles [90]. In another work by the same research group, PAN fabrics were grafted with GMA and chitosan. As shown in Figure 7, both the THR and the PHRR of the FR-PAN fibres demonstrated a reduction of 18.6% and 47.4%, respectively, when compared with the control samples. Moreover, the time to ignite increased by 36%, and the fire growth rate index decreased by 28.6%, indicating an enhancement in fire safety. Overall, the UV-induced graft coating provided the PAN fabrics with excellent flame resistance [134]. Liu et al. used UV radiation to graft GMA into the PAN chemical structure and then reacted with L-arginine (Agr) to add fire-resistance properties to it. Compared with neat PAN fibres, the PAN-g-GMA/Agr showed 51.6% of residual charts at 800 °C, and the PHHR and THR were significantly reduced by 36.3% and 8.1%, respectively, indicating an improvement in thermal stability. Furthermore, the grafted fabrics also showed a 53.6% increase in elongation at break and an LOI of 34.7%. The latter remained at 27% after 40 washing cycles, indicating excellent durability [135].

Using a different approach, Basuoni et al. used keratin to graft PAN fibres in order to improve a range of properties. The treated fibres demonstrated superior wettability, anti-static characteristics, and enhanced UV resistance compared to the non-treated fibres, all without compromising the strength of the fabric. Additionally, the final product maintained its washing durability, withstanding up to 20 cycles [136]. Wang et al. used L-cystine as a coupling agent to graft casein or collagen into the PAN structure to improve its ability to retain moisture. Their results showed that the modified fibres substantially increased moisture retention, increasing from 2.01% (neat PAN) to 6.08% with casein and 6.31% with collagen [137].

### 3.5. Carbonisation

Carbon fibres, known for their light weight, superior strength and modulus, and exceptional heat resistance, have been used as a reinforcement material across various sectors, including aerospace, automotive industries, and civil engineering [138]. PAN is the primary precursor for producing high-performance carbon fibres, accounting for nearly 90% of the global market share [139].

The transformation of PAN into carbon fibres is a thermal process involving three main steps: oxidative stabilisation, high-temperature carbonisation, and graphitisation. The initial stage, oxidative stabilisation, generally takes place at lower temperatures (200–300 °C) in an air setting. This stage forms a ladder-like structure, stabilising the precursor for higher-temperature processing. Afterwards, during the carbonisation stage, the fibres are subjected to heat in a nitrogen atmosphere up to 1600 °C. This process eliminates noncarbon atoms like hydrogen, nitrogen, and oxygen. Lastly, the fibres undergo graphitisation, where they are further heated (up to 2000 °C) in an inert atmosphere. This final stage improves the orientation of the basal planes and enhances the stiffness of the fibres [140].

**Figure 8:**
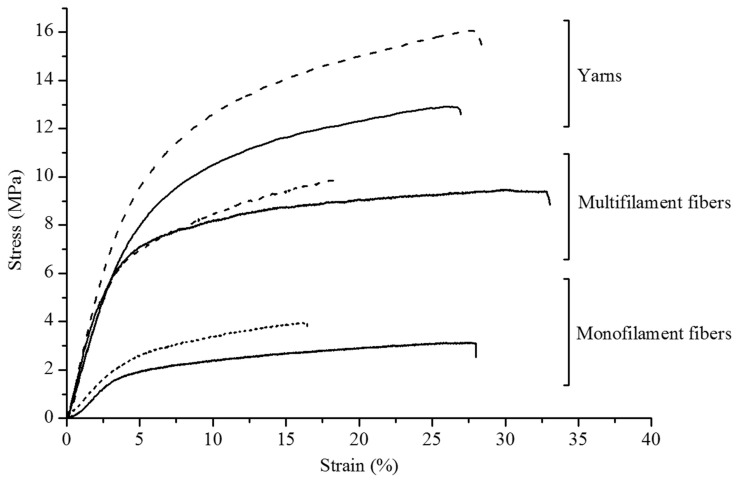
Stress–strain curves of PANb (solid) and PANb-CCG (dashed) fibres and yarns. Reproduced under the terms of the Creative Commons CC BY-NC-ND 4.0 license. Copyright 2019, the Authors [22]. Published by Willey.

A great deal of research has been conducted with the aim of improving the carbonisation of PAN fibres. Liu et al. developed a new precursor for PAN-based carbon fibres using 2-acrylamido-2-methylpropane acid (AMPS) and itaconic acid as the control. The P(AN-co-AMPS) nanofibres required less activation energy for cyclisation (E_a1_ = 26.6 kcal/mol and E_a2_ = 27.5 kcal/mol), and the carbon nanofibres derived from them showed a better graphite-like structure [138]. In another work, Xu et al. combined PAN with 1 wt.% of cellulose nanofibres (CNFs) through electrospinning and evaluated the carbonisation process. Their results indicated that the addition of CNFs enhanced the tensile strength and electrical conductivity of the PAN carbon nanofiber membrane. Furthermore, the presence of CNFs accelerated the carbonisation process and reduced the reaction requirements of the PAN nanofiber membranes [141]. Sayyar S et al. developed graphene/PAN-based fibres in the forms of monofilaments, multifilaments, and yarns using a wet-spinning technique. After that, the fibres were oxidised and carbonised at 900 °C to produce various types of carbon fibre samples (PANb-CCG). Their results showed that the addition of ~0.5 wt.% of graphene could increase the tensile strength and Young’s modulus of the PANb-CCG fibres by ~28% and ~20%, respectively. The improvement was more evident in multifilament and monofilament fibres (Figure 8), where the graphene could act as crosslinking points, helping to distribute stress uniformly throughout the fibres [22]. Sabantina et al. added varied concentrations of TiO_2_ nanoparticles into PAN fibres before carbonisation in order to apply carbon fibres to batteries or dye-sensitised solar cells. The final samples exhibited a fascinating pattern, whereby after electrospinning, a bead-like cluster structure of TiO_2_ was formed and became increasingly apparent along the fibres following stabilisation, especially after carbonisation. Moreover, greater concentrations of TiO_2_ appeared to reinforce the sample structure during the stabilisation process [142].

### 3.6. Dyeing Process

Dyes have been used by mankind for thousands of years, serving as essential materials in various aspects of life. From ancient times to the present day, the knowledge and application of dyes have evolved significantly. In terms of chemical structure, dyes can be classified as either organic or inorganic, where both categories can be further subdivided into natural or synthetic [143].

In the case of acrylic fibres, homo-PAN is highly resistant to dyeing due to its homopolymer’s dense structure and elevated glass transition temperature. The acrylic fibres with one or more comonomers instead are easier to dye, since these monomers reduce the structural regularity of the homopolymers, which reduces both the heat and solvent resistance and increases the fibres’ termoplasticity [144].

One way to dye acrylic fibres is through the use of disperse dyes. Disperse dyes are applied to acrylic fibres in the pH range between 3.5 and 6, usually in the presence of a levelling agent using dyeing procedures. Individual disperse dyes exhibit wide variations in dye uptake, with some providing much deeper and more brilliant shades, particularly through transfer printing techniques. Unfortunately, under normal dyeing conditions, disperse dyes are only suitable for achieving pale to medium shades. Better exhaustion can be achieved at higher temperatures, but 110 °C should be considered the maximum temperature due to the risk of excessive shrinkage in acrylic fibres at higher temperatures [144].

The presence of cyanide and acidic groups in the PAN structure can make them highly receptive to cationic dyes, due to their highly electronegative charge. When these fibres are immersed in a cationic dye bath, the dyes quickly bind to the anionic groups on the fibre surface. However, the dyeing process happens so fast that it often results in uneven dye distribution [145]. Typically, this issue is addressed by increasing the dye bath temperature or by adding surfactants, such as levelling agents. However, the addition of levelling agents (which increases wastewater discharge) and the rise in temperature makes the dyeing procedure less sustainable [146].

**Figure 9:**
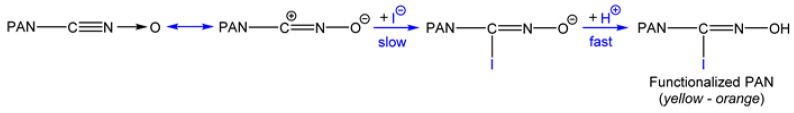
Polyacrylonitrile functionalised with iodine-oxime groups. Reproduced under the terms of the Creative Commons CC BY 4.0 license. Copyright 2021, the Authors [147]. Published by MDPI, Basel, Switzerland.

Trying to make the dyeing process more sustainable, Popescu et al. investigated the use of potassium hydroxide (KOH) and iodine-potassium iodide (I_2_/KI) to functionalise polyacrylonitrile (PAN) fibres and improve their tinctorial capacity. According to their results, KOH provided the necessary alkaline conditions for the functionalisation reactions to occur and increased the reactivity of the PAN fibres, facilitating the incorporation of new functional groups. Additionally, when combined with the I_2_/KI solution, it enabled the formation of iodine-oxime and iodinehydrin groups within the fibres, as shown by Figure 9. This process improved the dyeing properties of PAN fibres, allowing them to bond with a broader range of dyes, including acid dyes, such as C.I. Acid Red 57 and C.I. Acid Violet 48 [146]. Using a different approach, El Gabry et al. enhanced the transfer printability of acrylic fibres by treating them with sodium hydroxide, sodium polyacrylate, and bentonite nanocomposites. The acrylic fibres treated with sodium polyacrylate and sodium polyacrylate/bentonite nanocomposites showed higher colour intensity values than the untreated ones. According to the authors, the absorption improvement was due to the decrease in air permeability, which improved the absorption of the C.I. Disperse Red 60 dye vapours. Moreover, the nanobentonite addition also increased the fastness properties (washing, perspiration, and rubbing) of the acrylic fibres [29].

Dyes can also be used to give antimicrobial properties to acrylic fibres. Ma et al. utilised an exhaustion dyeing process to incorporate cationic dyes, composed of sodium sulphate and Triton X-100, into spun Orlan 75 (sulfonate-containing acrylic fabrics). The resulting treated fabrics demonstrated 99.9% effectiveness against *E. coli* and *S. aureus* proliferation before washing. However, the antibacterial efficacy diminished after 10 washing cycles, dropping to 45.5% [148]. Similarly, Abedi et al. explored a different dye composition by incorporating CI Direct Blue 168 with copper sulphate as a mordanting agent. The dyeing process consisted of three steps: pre-mordanting, dyeing, and post-mordanting. The pre and post samples with one percent and two percent of copper sulphate, respectively, exhibited the best antimicrobial activities, retaining an average efficacy of 96% even after eight washing cycles. Additionally, these fabrics showed minimal colour fading upon washing, showing the resilience of the dye solution [149]. Mehrizi et al. evaluated the antimicrobial performance of other dyes functionalised with copper and zinc sulphates, such as CI Direct Yellow 12, CI Direct Red 23, CI Direct Red 31, and CI Direct Black 38. The effectiveness of these fabrics varied significantly based on the chemical structure of the dyes. Notably, CI Direct Yellow 12 achieved the highest antimicrobial activity as well as good properties such as high molecular weight, washing fastness, linear structure, and ionic sulfonate groups [150].

## 4. Conclusions and Future Prospects

Acrylic-based fabrics are used in several applications due to their wool-like feeling and well-defined properties. Furthermore, through a range of processes, these fabrics can have their chemical structure modified, including the addition of new properties. However, many of the chemical substances used to perform these modifications have been banned because of their toxicity and health risks. Therefore, there has been a great deal of research and development into new sustainable materials.

Sol-gel and grafting technology are proving to be effective methods for substituting halogenated and formaldehyde-based products for flame-resistant finishing products. Using this method, some authors have achieved a substantial increase in the LOI values of their textile samples while maintaining their FR properties after various washing cycles. Another approach with promising results is the use of bio-based materials, such as chitosan, PVA, and hydrazine hydrate, with cases showing improvement in both the flame resistance and mechanical properties of the fibres.

In the case of water repellency, much effort has been made to substitute fluorocarbon-based compounds, especially PFOS and PFOA. Plasma treatment has been attracting significant attention as a new method to add water repellency properties to fabrics. Pre (cleaning) and post (fixing) treatments have been shown to improve the efficacy and durability of water-repellent coatings, with some results showing similarities with those of fabrics treated with traditional techniques.

Many types of fillers, such as silver, titanium, and carbon nanotubes, have demonstrably provided good UV and/or antimicrobial protection. For instance, some studies have shown that silver nanoparticles not only provide protection against *E. coli* and *S. aureus* (in some cases exceeding 90%) but are also able to significantly improve the UPF factor of PAN fabrics. Other particles, such as TiO_2_ and carbon nanotubes, have also exhibited promising results in terms of improving PAN UV protection, exceeding 100% in some cases. Nevertheless, other properties of such particles, such as conductivity, photocatalysis, and self-cleaning, are yet to be fully explored.

Moreover, thanks to a systematic investigation into the stabilisation and carbonisation of textile-grade PAN fibres, carbon fibres prepared from textile-grade PAN fibres were found to be comparable with those of commercial PAN fibres. Thus, PAN precursors from textiles could be an alternative for low-cost carbon fibre production, and this process could represent an interesting up-cycling option in the future.

Although finishing methods for acrylic textiles are becoming eco-friendly and more efficient, there is still a great deal of research to be done into alternatives to reduce the impact of these fibres, which are derived from petroleum. Furthermore, most acrylic fibres have not been recycled due to difficulties relating to the removal of contaminants from the textile. Therefore, more research needs to be done to remove chemicals from acrylic fabrics in order to break down the hazardous chemicals used during textile finishing into non-toxic substances, thereby promoting recycling.

## Figures and Tables

**Table 3:** Types of flame-retardant treatment methods and their LOI improvement efficiency of PAN fibres.

Author	Method	Char Residue in Air (%)	Char Residue in N_2_ (%)	LOI Improvement (%)	Ref.
Zhou et al.	Immersion in HHA and NaOH	46.3 ^a^	-	163	[82]
Zhou et al.	Fibre reinforcement with PVA and immersion in HHA and NaOH	-	-	53.2	[83]
Carosio et al.	Coating with chitosan and montmorillonite	12.0 ^b^	53.0 ^b^	10	[84]
Kim et al.	Copolymerisation of PAN with methyl caffeate	-	46.7 ^c^	24.8	[85]
Ren et al.	Sol-gel between PAN fibres and TEOS and urea	23.4 ^b^	55.9 ^b^	89.4	[86]
Yan et al.	Immersion in diethylenetriamine and zinc sulphate	-	37.5 ^a^	161.1	[87]
Dong et al.	Copolymerisation of PAN with dimethyl vinylphosphonate (DMVP)	44.9 ^a^	67.1 ^a^	61.3	[88]
Zhang et al.	Amidoximation using hydroxylamine hydrochloride (HA) followed by phosphorylation with phosphoric acid (PA)	-	55.7 ^b^	88.4	[89]
Ren et al.	UV grafting of PAN fibres with glycidyl methacrylate (GMA)	61.6 ^b^	-	90%	[90]
Zou et al.	Soaked in ammonium phytate solution followed by thermal oxidation	9.29 ^b^	72.35 ^b^	36.6	[91]

^a^ at 700 °C; ^b^ at 800 °C; ^c^ at 850 °C.
